# Transalveolar sinus floor lift without bone grafting in atrophic maxilla: A meta-analysis

**DOI:** 10.1038/s41598-018-19515-7

**Published:** 2018-01-23

**Authors:** Mingdong Yan, Ruimin Liu, Shuting Bai, Min Wang, Haibin Xia, Jiang Chen

**Affiliations:** 1 Department of Oral Implantology, Affiliated Stomatological Hospital of Fujian Medical University, Fuzhou, China; 2 The State Key Laboratory Breeding Base of Basic Science of Stomatology & Key Laboratory of Oral Biomedicine Ministry of Education, Wuhan University, Wuhan, China; 3 School of Stomatology, Fujian Medical University, Fuzhou, China; 4 Department of Oral Implantology, School and Hospital of Stomatology, Wuhan University, Wuhan, China

## Abstract

We performed a meta-analysis aimed to assess the clinical results after transalveolar sinus floor lift without bone grafting in the atrophic maxilla. A systematic electronic literature search was conducted in PubMed, Embase and The Cochrane Library, followed by a manual search. Two reviewers independently extracted study data and conducted quality assessments. Ten non-controlled studies including 1484 implants and eight controlled studies (5 RCTs and 3 prospective studies) including 817 implants (451 implants in the non-graft group) were enrolled in this study. The survival rate of implants via the graft-free method was 98% (95%CI 96% to 100%). There was no significant difference in the survival rate between the non-graft group and the graft group (RR: 1.02; p = 0.18). No statistically significant difference in marginal bone loss was detected between the groups at 12 months (0.57, p = 0.07) or 36 months (0.05, p = 0.61). The endo-sinus bone gain in the non-graft group was significantly lower than in the graft group at 12 months (−1.10, p = 0.0001) and 36 months (−0.74, p = 0.02). Hence, the available evidence suggests that predictable results could be acquired through transalveolar sinus floor lift without bone grafting, while there may be a trend toward more endo-sinus bone gain with bone grafts.

## Introduction

Endosseous dental implants have frequently been used to replace missing teeth and are considered a promising method for functional reconstruction in partially edentulous patients^1^. In the posterior maxilla, insufficient bone density and residual bone height due to bone resorption after tooth extraction or maxillary sinus pneumatization are usually observed. Such unfavourable conditions often cause difficulty in dental implant placement. Simultaneous bone grafting after a transalveolar maxillary sinus floor lift is one method to address these difficulties, and predictable results have been reported^2–4^.

However, there are some shortcomings of bone grafting after a maxillary sinus floor lift. Perforation of the Schneiderian membrane is the main complication of a sinus floor lift, and the incidence ranges from 12.5% to 44%^5,6^. Although no sufficient evidence suggests that perforation of the Schneiderian membrane reduces the survival rate, perforation in graft cases may cause the grafting materials to enter the sinus cavity, leading to inflammation^7,8^. In addition, the risk of immune rejection of the bone grafting material^9^ and higher costs are other disadvantages for transalveolar sinus floor lifts with bone grafting.

In the past decade, more researchers have focused on the clinical results of sinus floor lifts without bone grafts. Many studies have reported positive effects of sinus floor lifts without grafting^10–17^. However, different results have also been acquired in studies comparing graft with non-graft methods^18–24^. At present, the necessity of simultaneous bone grafting after a sinus floor lift is still controversial for researchers. Therefore, it is necessary to appraise the scientific literature on this topic. The aim of this meta-analysis was to assess the clinical results after transalveolar sinus floor lift without bone grafting in the atrophic maxilla. We conducted our study to assess three areas as follows: (1) identifying the survival rate of dental implants after sinus floor lift without grafting comparing the graft and non-graft groups; (2) investigating the survival rates of implants for different residual bone heights (RBHs) after transalveolar sinus floor lift without grafting; and (3) comparing the marginal bone loss (MBL) and endo-sinus bone gain (EsBG) between the two groups.

## Results

### Study identification and selection

**Figure 1:**
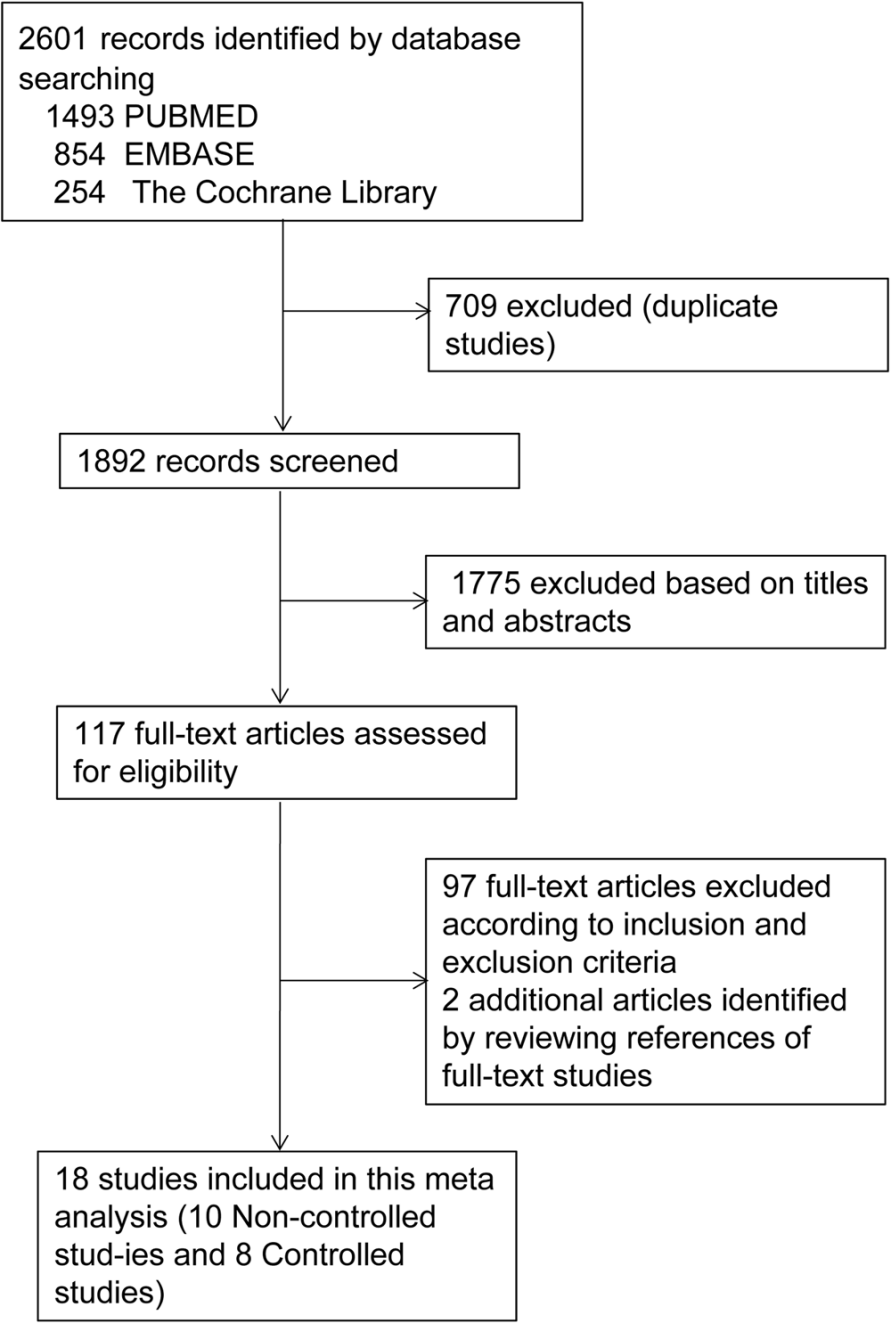
Flow diagram of study identification and selection.

The flowchart for study identification and selection is shown in Fig. 1. There were 2601 studies identified after the initial electronic search. Seven hundred and nine duplicate studies were excluded, and 1892 records remained. One thousand seven hundred and seventy-five articles were excluded after reviewing the titles and abstracts. The remaining 117 full-text articles were further evaluated for eligibility. Ninety-seven of them were also excluded due to not fulfilling the inclusion criteria or meeting the exclusion criteria, while two additional articles were identified by reviewing the references of the full-text studies. Ultimately, eighteen studies^10–27^, including 10 non-controlled studies and 8 controlled studies, were included in this meta-analysis.

### Characteristics of included studies

Among the fifteen included studies, there were 10 non-controlled studies assessing graft-free methods involving 1484 implants. Another 8 controlled studies comprised a total of 817 implants, including 451 implants in the non-graft group and 366 implants in the control group. The selected studies were published between 2008 and 2017 and were conducted in China, Canada, Spain, Sweden, Germany, Italy, Turkey and Switzerland. The main characteristics of the eighteen included studies are presented in Tables 1 and 2A. The outcome data for each included controlled trial are presented in Table 2B.

**Table 1:** Characteristics of non-controlled studies.

Study	Year	Study type	Country	Patients/Implant number	Inclusion Period	Implant Length (mm)	Initial RBH (mm)	Sinus elevation	EsBG (mm)	Follow-up (Months)	Survival rate	Measurement Instrument
Si MS	2016	RS	China	80/96	2006–2011	8 (n = 41) 10 (n = 47) 12 (n = 8)	6.75 ± 1.91	—	4 y: 2.95 ± 1.25 9 y: 2.16 ± 1.13	48–108	90.6%	PaR
Zill A	2016	RS	Germany	133/233	2001–2010	6 (n = 1) 8 (n = 4) 10 (n = 201) 12 (n = 27)	5.9 ± 1.7	—	4.5 ± 1.4	60	100%	PaR
French D	2016	RS	Canada	541/926	1998–2010	—	2-4 (n = 98); > 4 (n = 828)	—	—	4–120	97.0%	PR
Brizuela A	2014	PS	Spain	36/36	—	10.0 ± 1.0 10 (n = 32) 8 (n = 4)	7.4 ± 0.4 (Range: 4-9)	2.1 ± 0.3	1.8 ± 0.3	24	91.6%	PR
Gu YX	2016	PS	China	25/37	2007–2009	—	2.81 ± 0.74 (≤4)	—	—	60	94.6%	PR
He LL	2013	RS	China	22/27	2007–2009	10.0 ± 1.0 < 10 (n = 4) 10 (n = 19) > 10 (n = 4)	6.7 ± 1.2 (Range: 4.1-8)	4.1 ± 1.4	2.5 ± 1.5 (6 m)	25 ± 8	100%	CBCT
Fermergard R	2012	RS	Sweden	36/53	2003–2005	9 (n = 11) 11 (n = 34) 13 (n = 8)	6.3 ± 0.3	4.4 ± 0.2	—	36	94.3%	PR
Senyilmaz DP	2011	PS	Turkey	-/27	2007–2008	10 (n = 19) 8 (n = 8)	5–10	—	—	24	100%	PR
Nedir R	2010	PS	Switzerland	17/25	2003	10 (n = 21) 8 (3) 6 (1)	5.4 ± 2.3; ≤ 5 (n = 20); 5-8 (n = 5)	—	3.2 ± 1.3	60	100%	PaR
Schmidlin PR	2008	RS	Switzerland	24/24	2001–2004	10 (n = 9) 8 (n = 13) 6 (n = 2)	5.0 ± 1.5	2.6 ± 1.8*;2.8 ± 1.7^#^	2.2 ± 1.7*; 2.5 ± 1.5^#^	17.6 ± 8.4	100%	PR

PaR, periapical radiograph; PR, panoramic radiograph; CBCT, cone beam computed tomography; PS, prospective study; RS, retrospective study; *mesial aspect; ^#^distal aspect.

**Table 2:** (A) Characteristics of controlled studies. (B) Outcomes of controlled studies.

(A)												
Study	Year	Study type	Country	Inclusion Period	Patients	Initial RBH		ImplantLength (mm)	Graft materials in grafting group	Healing time (Months)	Measurement Instrument	Follow-up (Months)
						NG	G					
Lai HC	2010	PS	China	2003–2008	202	5.6 ± 2.5 mm	4.7 ± 2.1 mm	6/8/10	alloplastic bone-replacing material, β-tricalcium phosphate Cerasorb® mixed with autogenous bone chips	NG: 3-4 G:6-8	PaR, PR	Range: 12–60
Marković A	2016	RCT	Spain	2011–2012	45	6.59 ± 0.45 mm		10	β-tricalcium phosphate (β-TCP), deproteinized bovine bone (DBB), or their combination	6	CBCT	29.7
Nedir R	2013, 2016, 2017	RCT	Switzerland	2007–2009	12	2.6 ± 0.9 mm	2.2 ± 0.8 mm	8	inorganic bovine spongiosa bone mineral Bio-Oss®	2.6 ± 0.9	PaR	12, 36, and 60
Pjetursson BE	2009	PS	Switzerland	2000–2005	181	8.1 ± 2.1 mm	6.4 ± 1.9 mm	6/8/10/12	deproteinized bovine bone mineral Bio-Oss®	4–6	PaR	38.4 Range: 12–84
Si MS	2013	RCT	China	2007–2011	41	4.58 ± 1.47 mm	4.67 ± 1.18 mm	6/8/10	deproteinized bovine bone mineral (DBBM) mixed with autogenous bone chips	6	PR	36
Verdugo F	2017	PS	Italy	—	27	4.5 ± 0.8 mm	3.8 ± 1.2 mm	8/10/11.5	autogenous cortical bone particles	3–4	CBCT	Range: 36–144
**(B)**												
**Study**		**Year**	**Implant number**		**EsBG (mm)**				**MBL (mm)**		**Survival rate**	
			**NG**	**G**	**NG**		**G**		**NG**	**G**	**NG**	**G**
Lai HC		2010	191	89	—		—		—	—	97.38%	92.13%
Marković A		2015	45	135	—		—		—	—	100%	100%
Nedir R		2013	17	20	3.9 ± 1.0		5.0 ± 1.3		0.6 ± 0.8	0.4 ± 0.7	100%	90%
		2016			4.1 ± 1.0		5.1 ± 1.2		0.6 ± 1.1	0.5 ± 1.0	94.1%	90%
		2017			3.8±1.0		4.8 ± 1.2		0.6 ± 0.9	0.7 ± 1.4	94.1%	90%
Pjetursson BE		2009	164	88	1.7 ± 2		4.1 ± 2.4		—	—	97.56%	97.72%
Si MS		2013	20	21	6 months: 2.06 ± 1.01; 12 months: 2.45 ± 0.98; 24 months: 3.12 ± 0.70; 36 months: 3.07 ± 1.68		6 months: 5.66 ± 0.99; 12 months: 3.56 ± 1.82 24 months: 3.02 ± 0.48 36 months: 3.17 ± 1.95		6 months: 0.67 ± 0.92; 12 months: 1.28 ± 0.05; 24 months: 1.32 ± 0.45; 36 months: 1.38 ± 0.23	6 months: 0.21 ± 0.23; 12 months: 0.44 ± 0.16; 24 months: 0.65 ± 0.30; 36 months: 1.33 ± 0.46	95.0%	95.2%
Verdugo F		2017	14	13	6.8 ± 0.5		8.5 ± 1.9		0.9 ± 0.6	0.8 ± 0.7	100%	100%

PS, prospective study; NG, non-graft.

group; G, graft group; PaR, periapical radiograph; PR, panoramic radiograph; CBCT, cone beam computed tomography.

NG, non-graft group; G, graft group; MBL, marginal bone loss; EsBG, endo-sinus bone gain.

### Quality assessment of included studies

**Figure 2:**
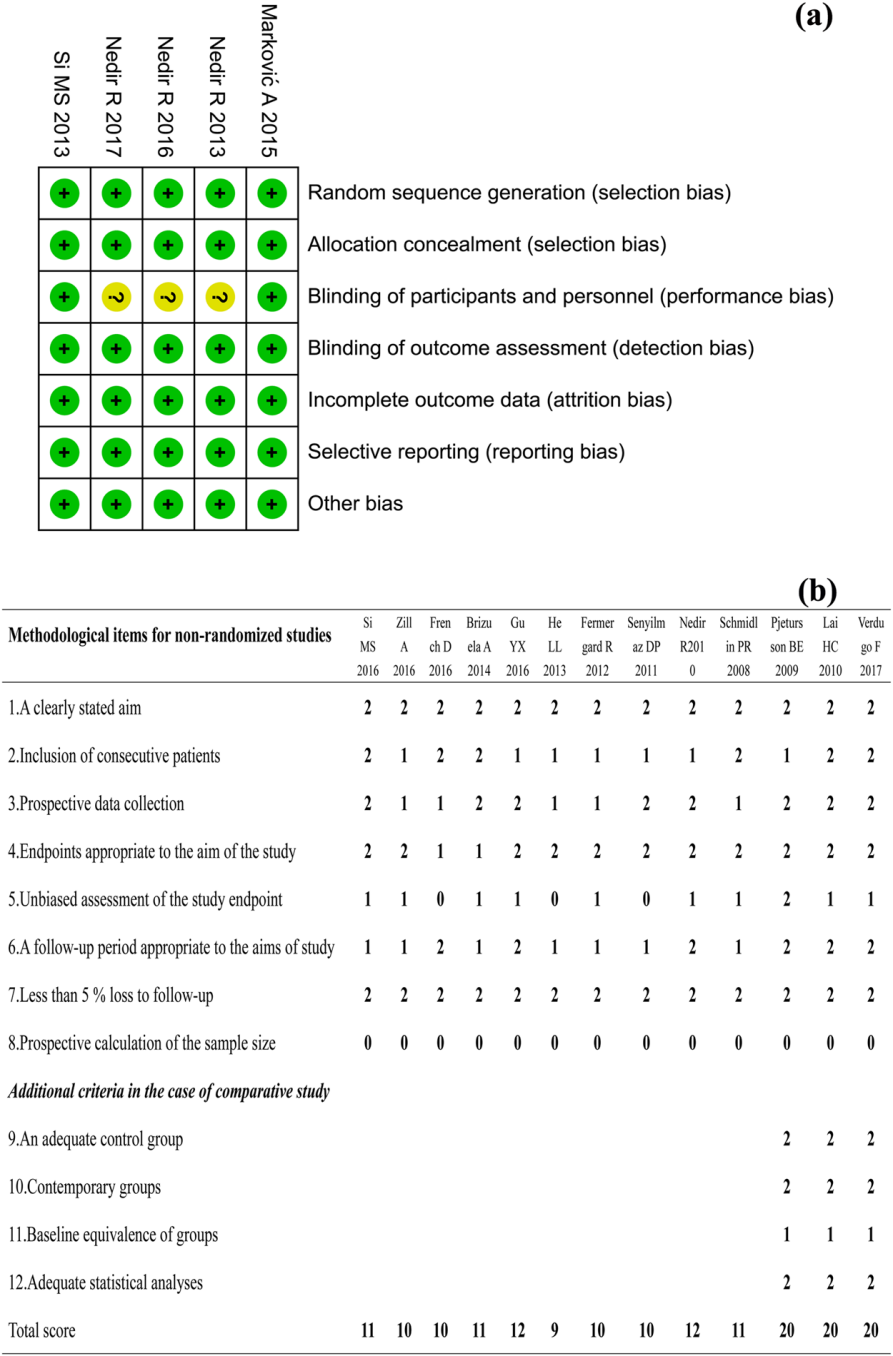
Quality assessment of included studies. (**a**) Risk of bias summary for randomized studies (“+” means low risk of bias, “?” means that the risk of bias is unclear, “−” means that the risk of bias is high). (**b**) Quality assessment for non-randomized studies by MINORS^31^.

We assessed the risk of bias of the 5 RCTs and 13 non-RCTs using a risk-of-bias assessment tool (Fig. 2).

For the 5 RCTs, the blinding of the participants and personnel was unclear in 3 trials. Two studies^22,23^ were identified as being “Low Risk Bias”. The risk of bias for the other 3 studies^19,20,24^ was unclear.

For the other 13 non-RCTs, all ten non-controlled studies acquired MINORS scores between 9 and 12. The remaining three uncontrolled studies acquired MINORS scores of 20. The methodological quality assessment is displayed in Fig. 2b.

### Analysis of outcomes

#### Primary outcomes

1. Survival rate of implants in non-controlled studies (without grafting)
  Survival rates for implants were available in all 10 included studies with the longest follow-up at 120 months. The meta-analysis was conducted using R software (version 3.1.3). As significant heterogeneity among the studies was detected (I^2^ = 78.8%, p < 0.0001), a random-effects model was selected for a more conservative effect. The pooled analysis showed that the survival rate of the graft-free method was 98% (95% confidence interval 96–100%) (Fig. 3a).

**Figure 3:**
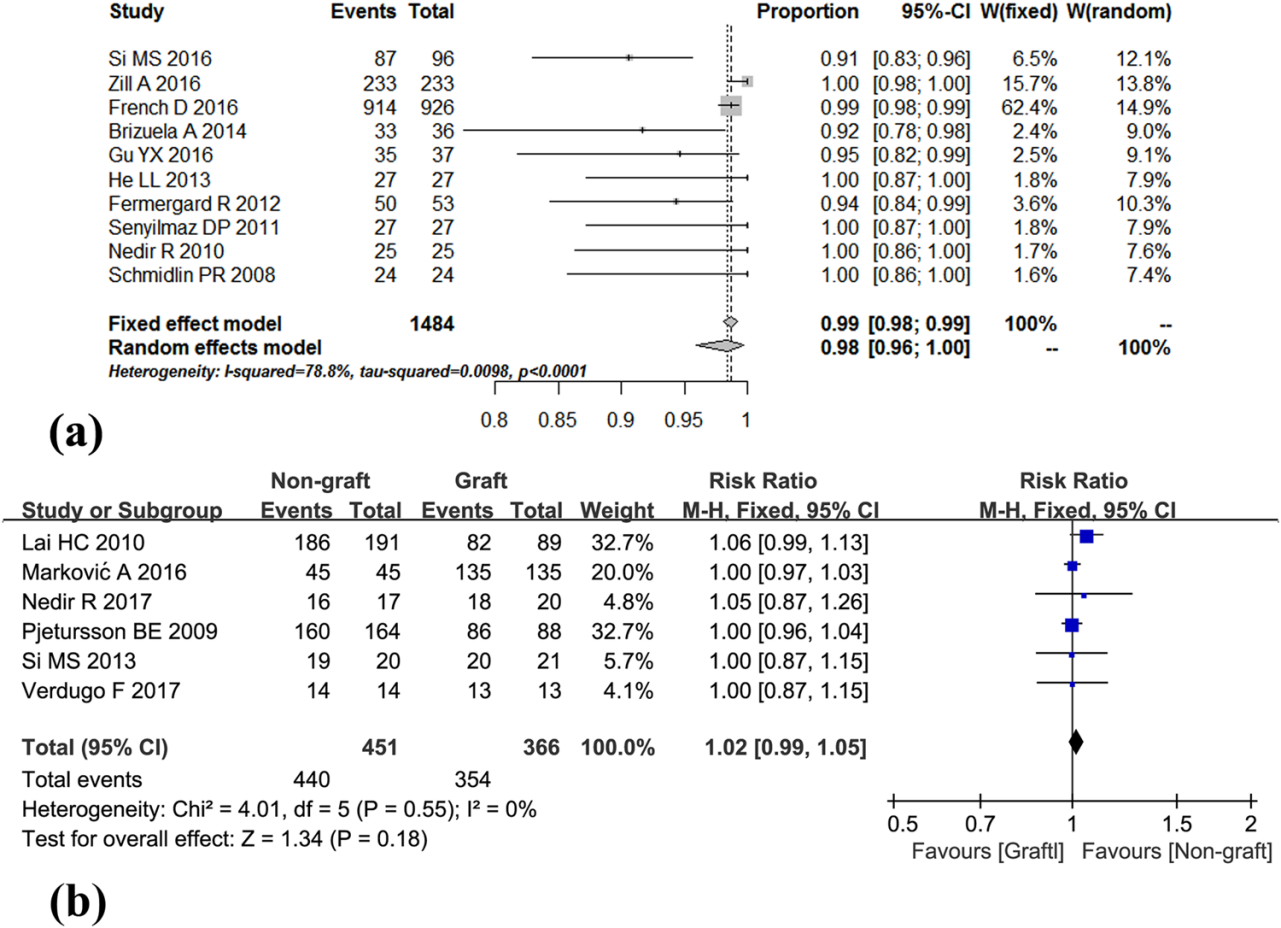
Forest plots of survival rates. (**a**) Forest plot of survival rate for non-controlled studies. (**b**) Forest plot of survival rate for controlled studies.
2. Survival rate of implants in controlled studies (non-graft group vs. graft group)
  Eight controlled studies, including 5 RCTs and 3 prospective studies, reported the survival rates in the non-graft group and graft group. As three RCTs^19,20,24^ included the same samples, we selected the longest follow-up data^24^. In the end, six studies were included in the pooled analysis. The pooled analysis was conducted using Review Manager software (version 5.2, Cochrane Collaboration, Oxford, UK). A risk ratio of 1.02 (95% confidence interval 0.99–1.05) was found in the meta-analysis. There was no statistically significant difference between the two groups (P = 0.18), and no heterogeneity among the studies was detected (I^2^ = 0%, p = 0.55) (Fig. 3b).
3. Survival rates of implants in different RBH for transalveolar sinus floor lift without grafting

Six of the ten non-controlled studies reported the RBH when the survival rates of the implants were available. Four studies included patients with RBH ≤ 4 mm and RBH > 4 mm. The survival rate at RBH ≤ 4 mm was 95.35%, while it was 96.34% for RBH > 4 mm. Two studies reported survival rates for patients with RBH ≤ 5 mm (95.18%) and RBH > 5 mm (95.12%) (Table 3).

**Table 3:** Survival rates of implants for different RBHs using a graft-free approach.

RBH (mm)	Included studies	Event/Implant number	Survival rate
≤4	Nedir R 2010/Gu YX 2016	41/43	95.35%
>4	Nedir R 2010/Brizuela A 2014/He LL 2013	79/82	96.34%
≤5	French D 2016/Si MS 2016	217/228	95.18%
>5	Senyilmaz DP 2011/French D 2016/Si MS 2016	781/821	95.12%

#### Secondary outcomes

1. Marginal bone loss
  Data on marginal bone loss at 12 months and 36 months were extracted from three studies^19,20,22^. No statistically significant difference was detected between groups at 36 months (0.05, 95%CI −0.16 to 0.27, p = 0.61), and no evidence of heterogeneity was found (I^2^ = 0.0%, p = 0.89) (Fig. 4a). The result at 12 months was 0.57 mm (95%CI −0.05 to 1.19, p = 0.07) with I^2^ = 84% (p = 0.01) (Fig. 4b).

**Figure 4:**
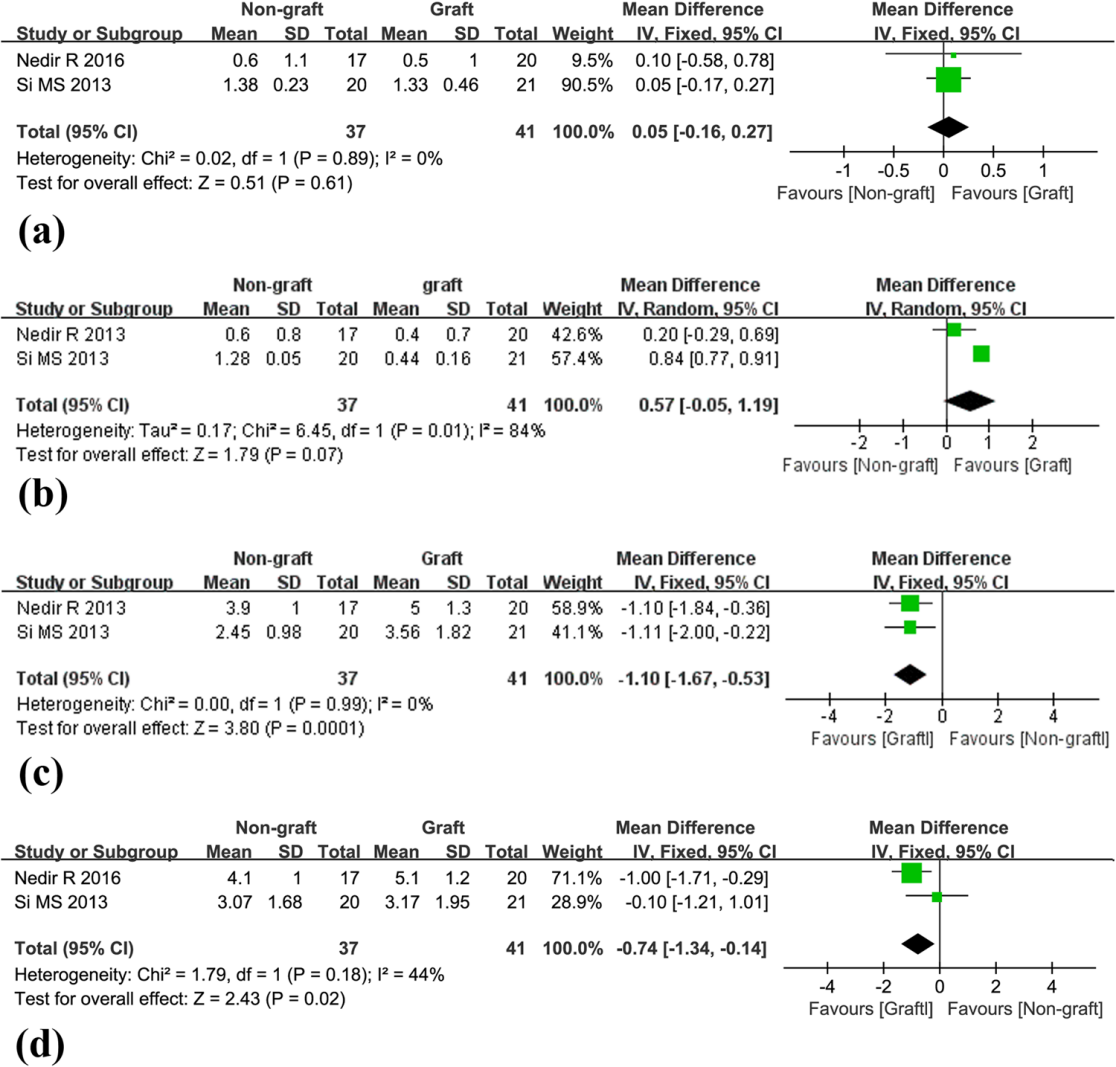
Forest plots of MBL and EsBG. (**a**) Forest plot of MBL at 36 months for the non-graft vs. the graft group. (**b**) Forest plot of MBL at 12 months for the non-graft vs. the graft group. (**c**) Forest plot of EsBG at 12 months for the non-graft vs. the graft group. (**d**) Forest plot of EsBG at 36 months for the non-graft vs. the graft group.
2. Endo-sinus bone gain

At 12 months, the endo-sinus bone gain in the non-graft group was significantly lower than in the graft group. This result amounted to a mean difference of −1.10 mm (95%CI −1.67 to −0.53, p = 0.0001; Fig. 4c), and no evidence of heterogeneity was detected (p = 0.99, I^2^ = 0.0%). A statistically significant difference was also observed at 36 months (−0.74, 95%CI −1.34 to −0.14, p = 0.02; Fig. 4d) with low heterogeneity across the studies (p = 0.18, I^2^ = 44%).

**Figure 5:**
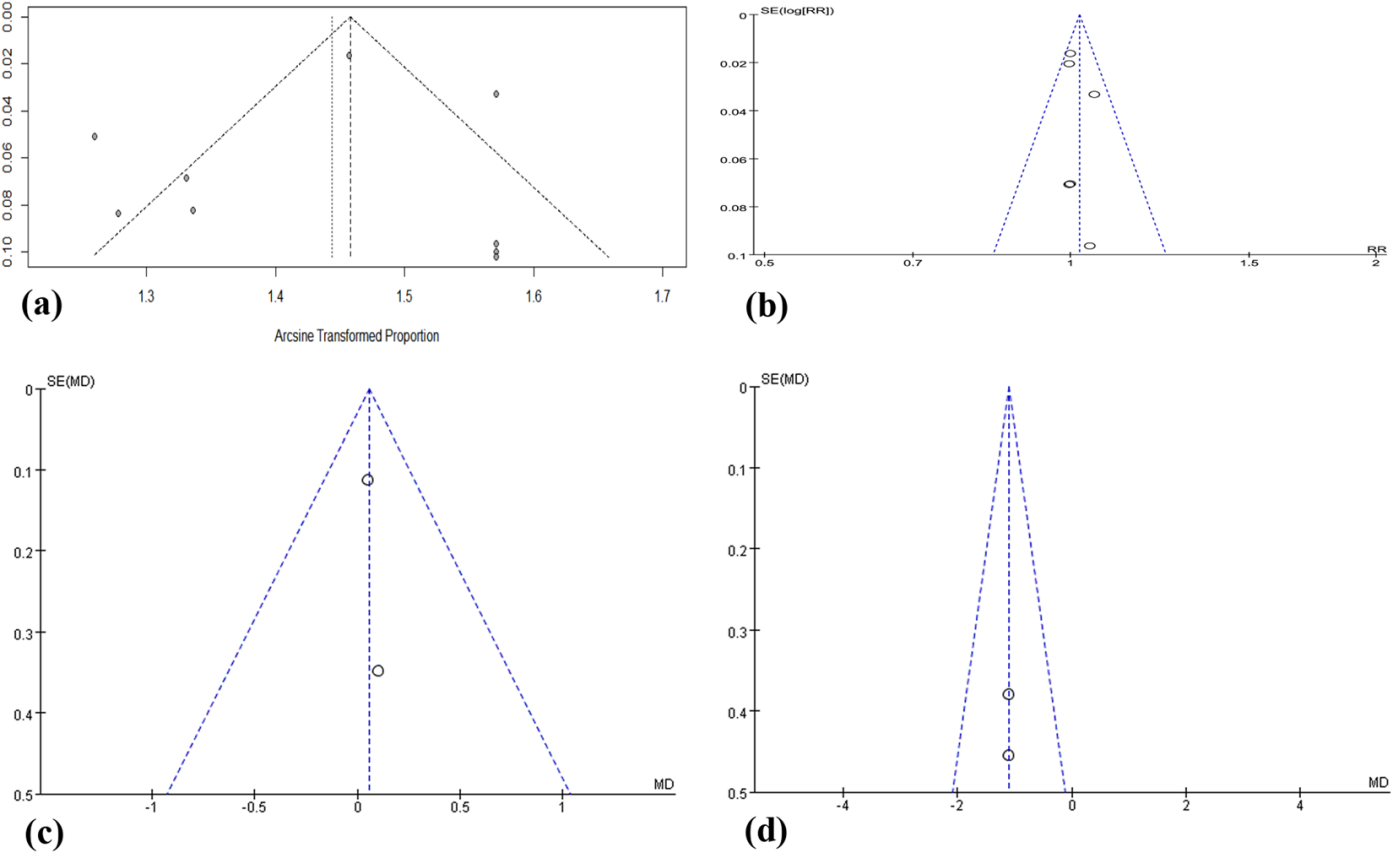
Funnel plots. (**a**) Funnel plot of survival rates in non-controlled studies. (**b**) Funnel plot of survival rates in controlled studies. (**c**) Funnel plot of MBL at 36 months. (**d**) Funnel plot of EsBG at 12 months.

Publication bias. No evidence of publication bias was detected, as all the outcomes had funnel plots with no significant asymmetry (Fig. 5).

## Discussion

Si MS *et al*.^22^ found a survival rate that was slightly higher in the graft group than in the non-graft group. Other researchers^18,21,24^ found that the survival rates in the graft group were slightly lower than in the control group. However, none of these studies showed a statistically significant difference in the survival rate between the two groups. The marginal bone loss at 12 months and the endo-sinus bone gain at 36 months showed statistically significant differences in different studies^19,20,22^. As most of these studies had small sample sizes, our meta-analysis might help to achieve more reliable results. To minimize the potential for bias, the reviewers extensively searched the published literature through a search engine (PubMed) and electronic databases (Embase and The Cochrane Library) and manually retrieved the references of the included studies. All the included studies were strictly enrolled based on the inclusion and exclusion criteria. Data extraction and quality assessment of the studies were performed by two reviewers, respectively. Ultimately, eighteen studies including five RCTs were included. The sample size for implants reached 1484 implants in the non-controlled studies and 817 implants in controlled studies. The results of our meta-analysis indicate that a graft-free sinus floor lift had a positive effect with a survival rate of 98% (95%CI 96–100%). This study showed good survival rates for implants in both cases with and without bone grafting after sinus floor lift. A slight survival benefit was detected in the non-graft group, but no statistically significant difference was observed (RR = 1.02, p = 0.18). The overall marginal bone loss at 12 months and 36 months was slightly higher in the graft group but the difference was again not statistically significant.

The studies included in our meta-analysis reported endo-sinus bone gain in the non-graft group, which was in accordance with prior studies suggesting that new bone could form under the lifted sinus membrane as a result of the physical space and blood clot formation^28,29^. The results of our meta-analysis suggest that the overall amounts of endo-sinus bone gain at 12 months and 36 months were lower in the non-graft group, and the difference was statistically significant. The differences were 1.10 mm at 12 months and 0.74 mm at 36 months.

There may be a trend for more endo-sinus bone gain when using grafting materials. Future studies could focus on the issue of whether this difference is the result of more bone resorption or simply less membrane elevation. Moreover, the graft shrinkage rate is also important. As only one study reported on post-OP X-rays in the graft group, a meta-analysis of the graft shrinkage rate could not be conducted.

Differences in the RBH might influence the survival rate of implants. The survival rate of implants for RBH ≤ 4 mm was only slightly lower than for RBH > 4 mm. However, different results were detected for RBH ≤ 5 mm compared with RBH > 5 mm, which could be attributable to the limited sample size.

Although no study reported a cost/effectiveness ratio, it is clear that the cost of a sinus floor lift without bone grafting must be lower than with grafting.

There are some limitations to this meta-analysis. First, certain issues that might potentially influence the results of the included studies should be clarified. The studies involve different study designs, initial RBHs, implant lengths, grafting materials, measurement instruments and implant types. In addition, the anatomical defect and need for grafting associated with surgical access and surgical technique may also influence the results of the included studies. Most studies measured outcome data by periapical radiograph or panoramic radiograph, while only three studie^14,23,27^ used cone beam computed tomography (CBCT). Second, there was a limited number of studies of different quality levels included. There were only 5 RCTs included, of which three^19,20,24^ used the same samples. Two RCTs had a low risk of bias, and three RCTs had a moderate risk of bias. The other studies were all non-RCTs. Although it is hard to quantify the influence of these risks of bias on the study results, such methodological shortcomings should be considered when interpreting the results of our study. Third, only articles published in English were included, which means that potentially relevant articles published in other languages might not have been identified. Excluding these studies might contribute as a potential source of bias in this study.

In conclusion, both graft-free sinus floor lifts and procedures with grafts have positive effects; the results of our meta-analysis indicate that there are no differences in survival rate and marginal bone loss between non-graft and graft groups; the endo-sinus bone gain in the graft group was slightly higher than in the non-graft group. However, because of the limitations of our study, as mentioned above, future well-designed RCTs with long-term follow-up are also required to substantiate our findings.

## Material and Methods

This study followed the guidelines of the PRISMA statement (http://www.prisma-statement.org/).

### Literature search

A systematic electronic literature search was conducted in PubMed, Embase and The Cochrane Library (all from inception to October 2017). The medical subject headings (MeSH) “sinus floor augmentation” and “bone transplantation” and the text words “sinus lift”, “sinus augmentation”, “sinus floor elevation”, “sinus elevation”, “bone graft*”, “graft*”, “bone augmentation”, “graft-free”, and “non-graft*” were used in combination with other strategies to identify potential studies. The publication language was restricted to English. To be as inclusive as possible, no limitations were set for the Design, Region, or Publication type. Moreover, a manual search of all the relevant references in the included studies was performed to discover other potentially eligible trials. This process was conducted iteratively until no additional trails could be identified. To minimize the potential for reviewer bias, two reviewers independently conducted electronic literature searches and performed study selection. The level of agreement between the reviewers was determined by the Cohen k test, assuming k = 0.61 as an acceptable agreement score^30^. Any disagreement regarding inclusion or exclusion of a retrieved study was resolved by discussion or consulting another reviewer.

### Inclusion and exclusion criteria

Studies that met the following criteria were eligible for inclusion:

1. Clinical studies assessing the clinical results after transalveolar or osteotome techniques to elevate the sinus floor without grafting, or RCTs comparing non-graft and graft groups;
2. Studies involving human adult subjects (age ≥ 18 years); and
3. Outcomes consisting of graft survival rates or EsBG or MBL.

The exclusion criteria were as follows:

1. Studies involving patients systemically contra-indicated for implant placement, affected by uncontrolled periodontal diseases or acute maxillary sinusitis;
2. Follow-up less than six months;
3. No outcome of interest;
4. Case report or review; or
5. Duplicate studies.

### Data extraction and outcome measurements

Data were independently abstracted by two reviewers using standardized tables that had been trialed prior to use. Any disagreement was resolved by discussion or consulting another reviewer. The primary outcome measurement was the survival rate of the implants, which was calculated as the ratio of the surviving implants to the total number of implants. The secondary outcome measurements included the endo-sinus bone gain (EsBG) and marginal bone loss (MBL). Other parameters, such as the relationship between the RBH and survival rate, were also evaluated. When any information was absent, the study authors were contacted. David French^10^ and Aleksa Marković A^23^ provided the detailed data of their published studies. When studies used the same samples but different durations of follow-up, the data for the longest follow-up period were extracted.

### Quality assessment

The two reviewers independently assessed the risk of bias for each included study, and all disagreements were resolved by consensus or consulting a third reviewer. RCTs were evaluated using the Cochrane Collaboration’s Risk of Bias tool (www.cochrane-handbook.org); when there was more than one item with “Unclear risk of bias” or “High risk of bias”, the quality of the study was considered “Unclear risk of bias” or “High risk of bias”. Non-RCTs (prospective studies and retrospective studies) were assessed using the Methodological Index for Non-Randomized Studies (MINORS)^31^. The methodological quality was scored from 0 to 16 for studies without a control group and from 0 to 24 for studies with a control group.

## Statistical analysis

Meta-analysis of the survival rate in non-controlled studies was performed using R software (version 3.1.3), while the other meta-analyses were conducted using Review Manager software (version 5.2, Cochrane Collaboration, Oxford, UK). We used the mean difference (MD) and risk ratio (RR) to compare continuous and dichotomous variables, respectively. All measures were reported with 95% confidence intervals (CIs).

Statistical heterogeneity was detected using the I^2^ statistic and the chi-squared test. First, a fixed-effects model was used. When significant heterogeneity (I^2^ > 50%) was detected, we changed to a random-effects model^32^. Subgroup analyses and sensitivity analyses were carried out when necessary to determine whether there was a difference in the results. Furthermore, the risk of publication bias was investigated for the included outcomes by analysing funnel plots through visual inspection^33^.
